# Identification of neoantigens in oesophageal adenocarcinoma

**DOI:** 10.1111/imm.13578

**Published:** 2022-10-19

**Authors:** Ben Nicholas, Alistair Bailey, Katy J. McCann, Oliver Wood, Robert C. Walker, Robert Parker, Nicola Ternette, Tim Elliott, Tim J. Underwood, Peter Johnson, Paul Skipp

**Affiliations:** ^1^ Centre for Proteomic Research, Biological Sciences and Institute for Life Sciences University of Southampton Southampton Hampshire UK; ^2^ Centre for Cancer Immunology and Institute for Life Sciences, Faculty of Medicine University of Southampton Southampton Hampshire UK; ^3^ School of Cancer Sciences, Faculty of Medicine University of Southampton Southampton Hampshire UK; ^4^ Centre for Cellular and Molecular Physiology, Nuffield Department of Medicine University of Oxford Oxford UK; ^5^ Centre for Immuno‐oncology, Nuffield Department of Medicine University of Oxford UK; ^6^ Cancer Research UK Clinical Centre University of Southampton Southampton Hampshire UK

**Keywords:** antigen presentation, HLA, oesophageal adenocarcinoma, peptidome

## Abstract

Oesophageal adenocarcinoma (OAC) has a relatively poor long‐term survival and limited treatment options. Promising targets for immunotherapy are short peptide neoantigens containing tumour mutations, presented to cytotoxic T‐cells by human leucocyte antigen (HLA) molecules. Despite an association between putative neoantigen abundance and therapeutic response across cancers, immunogenic neoantigens are challenging to identify. Here we characterized the mutational and immunopeptidomic landscapes of tumours from a cohort of seven patients with OAC. We directly identified one HLA‐I presented neoantigen from one patient, and report functional T‐cell responses from a predicted HLA‐II neoantigen in a second patient. The predicted class II neoantigen contains both HLA I and II binding motifs. Our exploratory observations are consistent with previous neoantigen studies in finding that neoantigens are rarely directly observed, and an identification success rate following prediction in the order of 10%. However, our identified putative neoantigen is capable of eliciting strong T‐cell responses, emphasizing the need for improved strategies for neoantigen identification.

## Graphical abstract

Oesophageal adenocarcinoma (OAC) has a relatively poor long‐term survival and limited treatment options. Here we characterized the mutational and immunopeptidomic landscapes of tumours from a cohort of seven patients with OAC. We directly identified one HLA‐I presented neoantigen from one patient, and report functional T‐cell responses from a predicted HLA‐II neoantigen in a second patient containing both HLA I and II binding motifs.

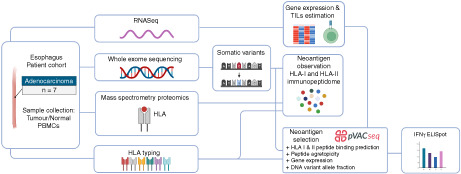

## INTRODUCTION

Oesophageal adenocarcinoma (OAC) is the 14th most common cancer in the UK, with a 10‐year survivability of 12% [1]. Early‐stage treatment of OAC involves resection of the oesophagus, whereas later stage diagnosis is treated with chemoradiotherapy or chemotherapy followed by surgery [2]. Relative to other cancers, OAC is characterized by having a high mutational burden, measured as the number of mutations per protein coding region [3]. Many of these mutations appearing in OAC driver genes [4, 5].

Tumour infiltrating lymphocytes (TILs), specifically cytotoxic CD8^+^ and CD4^+^ helper T‐cells recognize respectively, peptides of intracellular and extracellular origin presented by class I and II human leucocyte antigen (HLA) molecules. Presented at the cell surface, these HLA bound peptides form the immunopeptidome. Neoantigen peptides contain tumour mutations, making attractive therapeutic targets because of their potential to elicit tumour specific T‐cell responses.

Progress in developing neoantigen vaccines has been hindered by the difficulty in identifying neoantigen targets, and the challenge of overcoming the immunosuppressive tumour microenviroment [6]. In addressing neoantigen identification, direct identification using immunopeptidomics suggests observing neoantigens is rare [7]. Attempts to predict neoantigens using HLA binding algorithms show it is relatively straight forward to create a long list of potential putative neoantigens, but difficult to reliably select immunogenic neoantigens [8]. Large scale studies of OAC report the density of CD8^+^ T‐cells correlates with the number of somatic mutations [4], but an analysis of the OAC immunopeptidome is yet to be performed.

Here we explore a proteogenomics approach combining whole exome sequencing (WES), gene expression (RNASeq), HLA immunopeptidomics and algorithmic neoantigen prediction to identify neoantigens in seven OAC patients. We show that OAC has an abundance of somatic mutations and immunopeptides, and that whilst direct observation or prediction of immunogenic neoantigens remain challenging we were able to identify two neoantigens in two patients, one by direct observation and one by prediction. These findings are an important step towards demonstrating the usefulness of neoantigen based therapies for OAC.

## RESULTS

**FIGURE 1:**
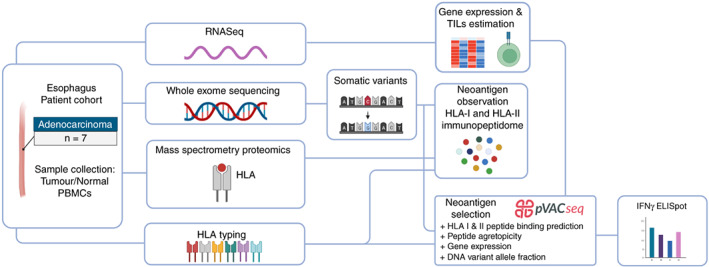
Workflow of the approach to identify human leucocyte antigen (HLA)‐I and ‐II neoantigens isolated from oesophageal tissues. IFN‐γ, interferon‐γ; PBMCs, peripheral blood mononuclear cells; TILs, tumour infiltrating lymphocytes.

We collected tissues comprising of tumour and adjacent normal tissue, and peripheral blood mononuclear cells (PBMCs) from seven male individuals with OAC (median age 68; Table 1). Whole genome sequencing for three donors have been previously deposited as part of ICGC project ESAD‐UK and EGA data set EGAD00001007785. We sequenced the exomes of tumour and normal tissues, and performed gene expression and immunopeptidomic analysis of the tumour tissues. PBMCs were used for HLA typing and interferon‐γ (IFN‐γ) ELISpot functional assays for patient EN‐181‐11 (Figure 1) [9].

**TABLE 1:** Clinical summary of patients in this study with oesophageal adenocarcinoma

Donor	ICGC donor	Age at diagnosis	Sex	Tumour location	Tumour stage	Treatment modality
EN‐181‐11	DO234382	64	Male	Gastro‐oesophageal junction (Siewert II)	2	Surgery
EN‐216‐11	DO50307	74	Male	Lower oesophagus	2	Surgery
EN‐430‐11		78	Male	Lower oesophagus	3	Chemotherapy + surgery
EN‐454‐11	DO50387	81	Male	Gastro‐oesophageal junction (Siewert II)	3	Surgery
EN‐489‐12		62	Male	Lower oesophagus	2	Chemotherapy + surgery
EN‐711‐16		68	Male	Lower oesophagus	3	Chemotherapy + surgery
EN‐716‐11		66	Male	Lower oesophagus	3	Chemotherapy + surgery

Abbreviation: ICGC, International Cancer Genome Consortium.

### The mutational landscape of seven OACs

**FIGURE 2:**
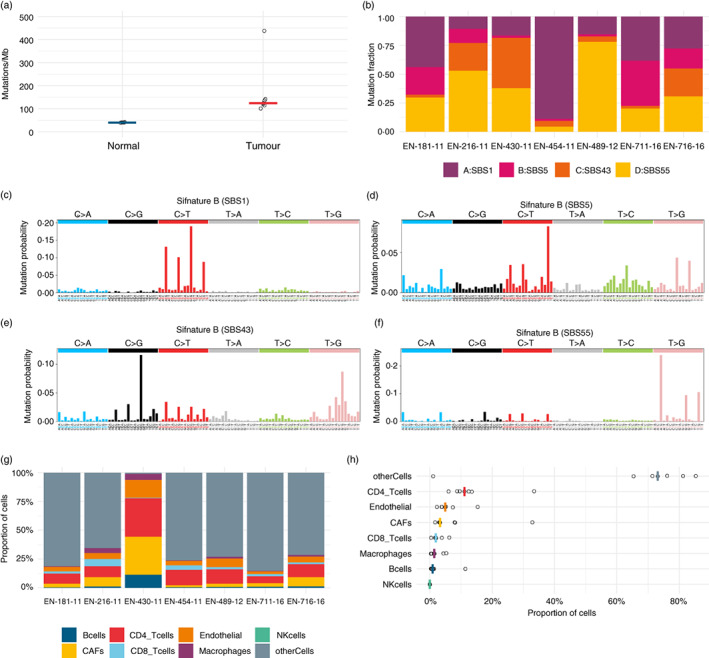
The mutational landscape of seven oesophageal adenocarcinomas (OACs). (a) The mutational burden of tumour and normal adjacent tissues from whole exome sequencing (WES) assuming a whole exome size of 30 Mb. The bar demarks the median. (b) The proportions of the four single base substitution mutational signatures in each OAC sample extracted from WES. The best fit signatures in COSMIC v3 database are prefixed Single base substitution. (c–f) The four mutational signatures extracted from WES of seven OAC samples. (g) The proportions of tumour infiltrating lymphocytes (TILs) estimated from bulk tumour RNASeq in each OAC sample. (h) The proportions of TILs estimated from bulk tumour RNASeq across the cohort. The bar demarks the median

To assess the likelihood of identifying HLA presented neoantigens we first examined the mutational landscape of the seven OACs. Somatic mutations accumulate in the genome over time as cells divide, and in cancer the causes and patterns of somatic mutations help characterize the cancer type and explain its cells selective advantage [3]. The total number of somatic mutations per coding region of genome defines the mutational burden of the cancer type, and has been correlated with response to anti‐Programmed cell death protein 1 therapy, and is therefore a proxy for the number of neoantigens presented by tumour cells [10, 11]. Across cancers, median mutational burden ranges from 16 to over 300 mutations per megabase [3]. Here, the OACs had a median mutational burden of 124 mutations/Mb in comparison to a median of 40 mutations/Mb for normal adjacent tissue (Figure 2[a], Table S1). Four patterns of single base substitutions created by the somatic mutations were extracted as mutational signatures and fitted to those identified in COSMIC [12, 13, 14] (Figure 2[b–f]). Different fractions of these four signatures were seen in each individual (Figure 2[b]). Extracted signatures A and B contain high proportions of C > T substitutions, fitting signatures SBS1 and SBS5, respectively (Figure 2[c,f]). These are both clock like signatures correlated with ageing and have previously been reported in large scale studies of OAC [4, 13]. Extracted signatures C and D fit signatures SBS43 and SBS55 respectively, and contain high proportions of T > G substitutions (Figure 2[a,c]). COSMIC flags these signatures as possible sequencing artefacts, but high proportions of T > G substitutions have been reported as characteristic of OAC, and indicative of high levels of neoantigen presentation [4].

The immune response to neoantigens is also contingent on the ability of immune cells to infiltrate tumours. Using bulk tumour gene expression data, we estimated the fraction of TILs present in our tumours [15] (Figure 2[g,h]). This estimation also indicates a measure of tumour purity by collating all the genes not corresponding with TILs as ‘otherCells’, which we would expect to make up the majority of the cells in a tumour sample. Therefore the estimation of only 1% other cells for EN‐430‐11 as compared with a median of 73% indicates that the biopsy captured predominantly non‐tumour material. The remaining six samples have similar proportions of CD4^+^ and CD8^+^ TILs, with median fractions of 11% and 2% respectively, indicating the presence of TILs, a necessary but not sufficient requirement for a response to presented neoantigens.

In summary, the mutational landscape of our OAC samples is characterized by a high tumour mutational burden along with the presence of TILs, both necessary conditions for neoantigen presentation and recognition respectively.

### Immunopeptidome analysis reveals one putative neoantigen

**FIGURE 3:**
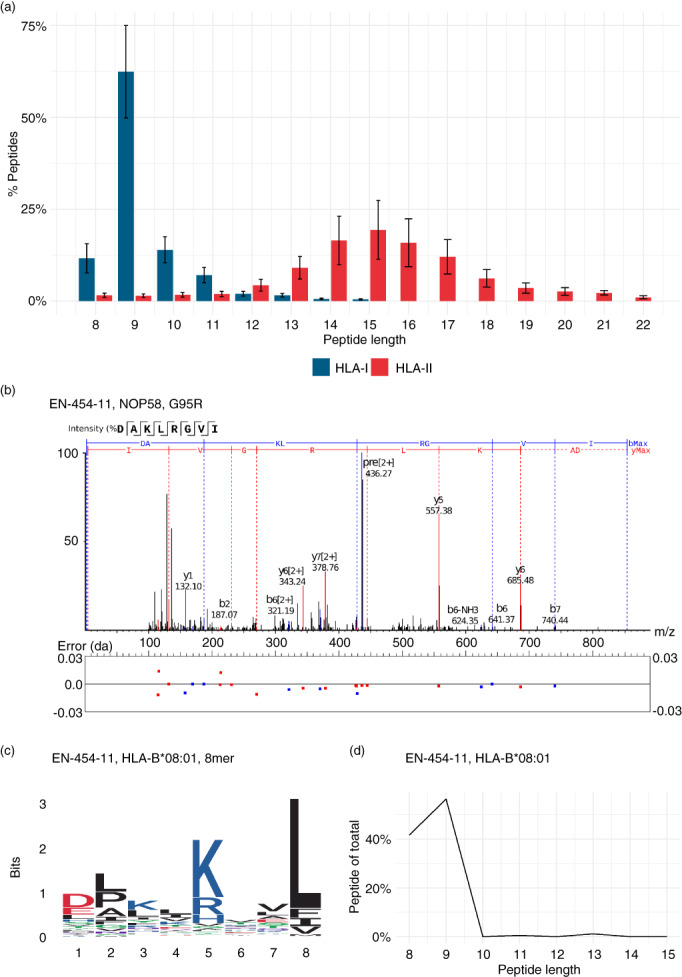
A single putative neoantigen identifed from the immunopeptidomes of seven oesophageal adenocarcinomas (OACs). (a) Histogram of 41 535 eluted human leucocyte antigen (HLA) class I and II peptides from seven OAC samples. (b) MS/MS spectrum from donor EN‐454‐11 of putative HLA‐B*08:01 8‐mer neoantigen DAKLRGVI. (c) Motif of all 8‐mer peptides from donor EN‐454‐11 assigned to HLA‐B*08:01. (d) Length distribution of *n* = 650 peptides from donor EN‐454‐11 assigned to HLA‐B*08:01

We next sought to directly observe neoantigens present in the immunopeptidomes of our OAC samples. Using the mutations identified from WES we created individual databases appended with patient specific mutated sequences (mutanomes) to search for neoantigens in their immunopeptidomes (Figure 1). In total we identified 41 535 HLA class I and II peptides isolated from these tumours by Liquid Chromatography with tandem mass spectrometry analysis at a false discovery rate (FDR) of 1% (Table S1). These immunopeptidomes comprised of 24 095 unique class I and 8023 unique class II peptides, forming characteristic HLA length distributions with modes of 9‐mers and 15‐mers for HLA class I and II peptides, respectively (Figure 3[a]).

Across the seven patients we identified only one putative neoantigen from the HLA‐I immunopeptidome of patient EN‐454‐11 (Figure 3[b]) [16]. This is an 8‐mer peptide derived from Nucleolar protein 58 (Gene: NOP58, UniProt: Q9Y2X3 COSMIC: COSV51895876) with a hydrophobic glycine to basic arginine mutation at protein amino acid (AA) residue 95, peptide AA residue 5. The mutation at peptide residue 5 creates a sequence DAKLRGVI with anchor residues for HLA‐B*08:01 (Figures 3[c], S1) [17, 18]. Over 40% of the 650 HLA‐B*08:01 peptides identified for EN‐454‐11 were 8‐mers, consistent with previous reports of a secondary length preference for 8‐mers for this allotype (Figure 3[d]) [18]. Unfortunately, there were insufficient PBMCs available to perform functional assays for this donor, so we next focused on predicted neoantigens from patient EN‐181‐11 from which we could perform a functional assay.

### Neoantigen prediction and functional analysis identifies a neoantigen with both class I and II HLA motifs

Neoantigen predictions from the EN‐181‐11 mutanome of 8–11mer peptides for class I HLA‐A and B, and of 15‐mer peptides for class II DRB1 allotypes were calculated using pVACseq [19, 20]. Neoantigen rank score is calculated as a function of the predicted binding affinity, the neoantigen agretopicity (the relative increase in predicted binding affinity of mutant peptide to wildtype peptide), the variant allele frequency and gene expression levels (Figure 1, Section 4). Predictions were performed for each peptide length and allotype combination yielding 15 ranked tables, comprising a total of 6842 peptides with binding affinity <500 nM for patient EN‐181‐11. Nine top ranking putative neoantigens were selected for functional analysis (Table 2, Supporting Information).

**TABLE 2:** Summary of top EN‐181‐11 predicted neoantigens

Gene	WT peptide	MT peptide	Mutation	Length	HLA allotype	WT (nM)	MT (nM)	Agretopicity	Gene expression (tpm)	VAF	Rank
ARAP2	KNFITQKYK	KSFITQKYK	N/S	9	HLA‐A*03:01	590	60	10	13	0.47	2
LAMB1	LVRFFRAPL	RVRFFRAPL	L/R	9	HLA‐B*07:02	40	12	3	98	0.08	1
LAMB1	LVRFFRAPL	RVRFFRAPL	L/R	9	HLA‐B*08:01	141	217	1	98	0.08	2
BRD8	LLPTSPRLVN	LLPTSPRLVK	N/K	10	HLA‐A*03:01	9940	74	134	98	0.16	3
ACTN1	QIAAIAQELN	QIAAIAQELK	N/K	10	HLA‐A*03:01	16 632	215	77	127	0.09	4
ZNF587	IQHQRVHTGQ	IQHQRVHTGK	Q/K	10	HLA‐A*03:01	39 477	355	111	78	0.15	5
TSC22D1	SHVAVASASI	SPVAVASASI	H/P	10	HLA‐B*07:02	5496	93	59	169	0.09	1
SMC1A	HRYVRGKSNL	HPYVRGKSNL	R/P	10	HLA‐B*07:02	17 442	144	121	105	0.07	2
WNK1	SHSSTTGLAF	SPSSTTGLAF	H/P	10	HLA‐B*07:02	8724	43	205	22	0.08	4
COL12A1	TLYLNVTDLKTYQIG	TLYLIVTDLKTYQIG	N/I	15	DRB1*03:01	406	45	9	54	0.67	1

*Note*: nM is median value of eight class I or four class II algorithms.

Abbreviations: HLA, human leucocyte antigen; MT, mutant; tpm, transcripts per million; VAF, variant allele frequency; WT, wildtype.

**FIGURE 4:**
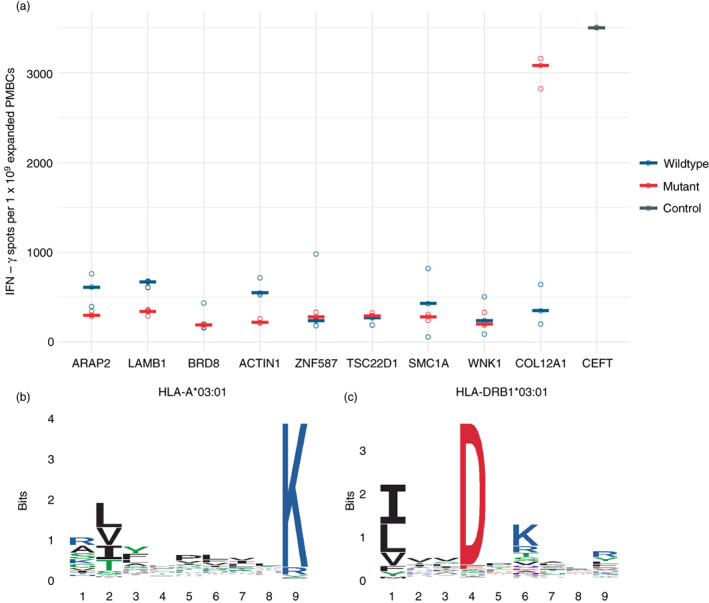
Functional T‐cell assay identifies a responding neoantigen containing class I and II human leucocyte antigen (HLA) motifs. (a) Interferon‐γ ELISpot of nine predicted neoantigen peptides (mutant) and their wildtype equivalents. Lengths and sequences as provided in Table 2. (b) HLA‐A*03:01 9‐mer motif assigned to EN‐181‐11 observed peptides. (c) HLA‐DRB1*03:01 core motif assigned to EN‐181‐11 observed peptides. PBMCs, peripheral blood mononuclear cells.

We synthesized both the neoantigen and wild type peptides at their specific lengths (Table 2) and tested their ability to stimulate T‐cells present in PBMCs using an IFN‐γ release cultured ELISpot assay (Figure 4[a]). We observed a strong response for only the putative class II neoantigen derived from collagen alpha‐1(XII) chain (Gene: COL12A1 UniProt: Q99715). Closer examination of the COL12A1 neoantigen sequence revealed that the first nine amino acids TLYLIVTDLK contain the HLA‐A*03:01 motif in addition to the HLA‐DRB1*03:01 motif in the full length TLYLIVTDLKTYQIG peptide (Figures 4[b,c], S2 and S3). Moreover, the observation of COL12A1 wild type peptides in both the class I and II immunopeptidomes of EN‐181‐11 indicate that this protein is presented in both antigen processing pathways by this tumour (Supporting Information).

## DISCUSSION

Here we report the first in‐depth study of HLA presented neoantigens in OAC, investigating both direct observation and predicted neoantigens from seven patients.

The mutational landscape of these OAC patients described by WES is consistent with previous characterizations of high mutational burden [14], mutational signatures with high proportions of C > T substitutions and evidence of chromosomal instability [4, 5, 21]. Gene expression analysis estimating the populations of infiltrating lymphocytes indicated that mutations yielding neoantigens may be detectable. However, only one neoantigen could be identified following direct examination of neoantigens using mass spectrometry‐based proteomics to identify HLA bound peptides eluted from tumour tissues. This is consistent with previous attempts at direct neoantigen identification across multiple cancer types [7, 22, 23].

Although we were unable to validate the functionality of this observed neoantigen due to unavailability of PBMCs for this individual, the observed neoantigen had an optimum length and binding motif for one of the patients HLA molecules. The G > R mutation changes this peptide from a wildtype peptide that would not be expected to bind to HLA and therefore not be presented, to a peptide that can bind and be presented. Hence, we believe that this is likely to be a true neoantigen, although we are unable to confirm if it is also immunogenic.

Evidence from checkpoint inhibitor therapy and T‐cell responses to predicted neoantigens suggests that neoantigens are effective at eliciting immune responses [24, 25, 26]. Therefore, for another patient for which there was available PBMCs we used the mutational and gene expression information to generate ranked lists of predicted neoantigens for each HLA‐A, B and HLA‐DRB1 allotype [19, 20]. We tested nine of the top ranked predicted neoantigens and their wildtype equivalents for their ability to stimulate T‐cells in an IFN‐γ release cultured ELISpot assay and found a single high responding neoantigen (>3000 spots/million cells, the wildtype peptide did not respond.) As with our attempts at direct identification, a one in nine success is comparable with previous reported attempts at predicting functional neoantigens [8]. The responding 15‐mer peptide was a HLA‐DRB1 predicted neoantigen, but on examination the first nine amino acids also comprised a HLA‐A neoantigen for this patient.

The identification of a neoantigen containing both HLA‐I and HLA‐II motif corresponds with reports of primarily CD4 responsive neoantigens even where neoantigens have been predicted as HLA‐I peptides eliciting CD8 responses [25, 26]. Similar observations have been reported in studies for viral pathogen specific peptides of CD4 responses where CD8 responses would be expected to predominate [27]. A feature of many neoantigen studies is the use of long peptides containing the neoantigen sequence and the reliance on cellular machinery to process these 20–25mer peptides into appropriate HLA‐I or HLA‐II length neoantigens [26, 28]. Without further deconvolution, such as pre‐enrichment for CD8 T‐cells prior to ELISpot or single cell RNAseq T‐cell receptor analysis, it is unclear what peptide processing has occurred and what immune response is being observed [29]. Here we used the specific peptides, but there still remains uncertainty about whether this a combined CD4/CD8 response or only CD4 response. Likewise the reasons for reports of predominantly CD4 responses, especially to HLA‐I ligands, remain unclear.

The main limitation of our study was the availability of PBMCs for validation of putative neoantigens. Our identification of a functional neoantigen in one patient suggests that we would be able to identify others across the cohort if we were able to test them.

Overall, this study confirms that either direct observation or prediction of functional neoantigens is rare with existing methodologies, and thus further work is required to increase the frequency of successful identification [29]. However, our study also demonstrates that identified neoantigens can yield strong immune responses in functional assays, highlighting the potential for the development of neoantigen based T‐cells vaccines and expanding the treatment options for a cancer with low survivability.

## MATERIALS AND METHODS

### Ethics statement

Informed written consent was provided for participation by all individuals. Ethical approval for this study was granted by the Proportionate Review Sub‐Committee of the North East – Newcastle & North Tyneside 1 Research Ethics Committee (Reference 18/NE/0234). This study was approved by the University of Southampton Research Ethics Committee.

### Tissue preparation

Seven subjects diagnosed with OAC were recruited to the study (see Table 1 for clinical characteristics). Tumours were excised from resected oesophageal tissue post‐operatively by pathologists and processed either for histological evaluation of tumour type and stage, or snap frozen at −80°C. Whole blood samples were obtained, and PBMCs were isolated by density gradient centrifugation over Lymphoprep prior to storage at −80°C.

### HLA typing

HLA typing was performed by Next Generation Sequencing by the NHS Blood and Transplant Histocompatibility and Immunogenetics Laboratory, Colindale, UK.

### DNA and RNA extraction

DNA and RNA were extracted from tumour tissue that had been obtained fresh and immediately snap frozen in liquid nitrogen. Ten to twenty 10 μm cryosections were used for nucleic acid extraction using the automated Maxwell® RSC instrument (Promega) with the appropriate sample kit and according to the manufacturer's instructions: Maxwell RSC Tissue DNA tissue kit and Maxwell RSC simplyRNA tissue kit, respectively. Similarly, DNA was extracted from snap frozen normal adjacent oesophagus tissue as described above. DNA and RNA were quantified using Qubit fluorometric quantitation assay (Thermo Fisher Scientific, UK) according to the manufacturer's instructions. RNA quality was assessed using the Agilent 2100 Bioanalyzer generating an RNA integrity number (Agilent Technologies UK Ltd.).

### Whole exome sequencing

The tumour and normal adjacent samples were prepared using SureSelect Human All Exon V7 library (Agilent, Santa Clara, USA). The 100 bp paired end reads sequencing was performed using the Illumina NovaSeq 6000 system by Edinburgh Genomics (Edinburgh, UK) providing ~100× depth. Reads were aligned to the 1000 genomes project version of the human genome reference sequence (GRCh38/hg38) using the Burrows–Wheeler Aligner (version 0.7.17) using the default parameters with the addition of using soft clipping for supplementary alignments. Following Genome Analysis Toolkit (GATK) Best Practices, aligned reads were merged [30], queryname sorted, de‐duplicated and position sorted [31] prior to base quality score recalibration [32].

### Somatic variant calling

Somatic variant calling was performed using three variant callers: Mutect2 (version 4.1.2.0) [33], VarScan (version 2.4.3) [34] and Strelka (version 2.9.2) [35]. For Mutect2, a panel of normals was created using 40 samples (20 male and 20 female) from the Great Britain dataset. Variants were combined using GATK ( GenomeAnalysisTK [version 3.8‐1]) with a priority order of Mutect2, VarScan, Strelka. Variants were then left aligned and trimmed, and multi‐allelic variants split [36]. Hard filtering of variants was performed such that only variants that had a variant allele fraction >5%, a total coverage >20 and variant allele coverage >5 were kept. Filtered variants were annotated using VEP (version 97) [37] and with their read counts (https://github.com/genome/bam-readcount) to generate the final filtered and annotated variant call files (VCF).

### RNA sequencing

Samples were prepared using TruSeq unstranded mRNA library (Illumina, San Diego, CA) and paired sequencing was performed using the Illumina NovaSeq 6000 system by Edinburgh Genomics. Raw reads were pre‐processed to use fastp (version 0.20.0) [38]. Filtered reads were aligned to the 1000 genomes project version of the human genome reference sequence (GRCh38/hg38 using hisat2 [version 2.1.0]) [39], merged and then transcripts assembled and gene expression estimated with StringTie2 (version 1.3.5) [40] using reference guided assembly.

### Mutanome generation

The annotated and filtered VCFs were processed using Variant Effect Predictor (version 97) [37] plugin ProteinSeqs to derive the amino acid sequences arising from missense mutations for each sample for use in immunopeptide analyses.

### Neoantigen prediction

VCF were prepared for the pVACseq neoantigen prediction pipeline (version 1.5.1) [19, 20] by adding tumour and normal DNA coverage, and tumour transcript and gene expression estimates using VAtools (version 4.1.0) (http://www.vatools.org/). VCF of phased proximal variants were also created for use with the pipeline [41]. Prediction of neoantigens arising from somatic variants was then performed using pVACseq with the patient HLA allotypes to predict 8–11mer peptides for class I HLA and 15‐mer peptides for class II HLA‐DRB allotypes. Eight binding algorithms were used for class I predictions (MHCflurry, MHCnuggetsI, NNalign, NetMHC, PickPocket, SMM, SMMPMBEC, SMMalign) and four for class II predictions (MHCnuggetsII, NetMHCIIpan, NNalign, SMMalign). Unfiltered outputs were post‐processed in R [42] and split into individual tables for each peptide length and HLA allotype for each patient, and each table was then ranked according to the pVACseq score, where:

$$\text{score}=\text{binding score}+\text{fold change}+\left(\text{variant expression}\times \text{fold change}\right)+\left(\text{tumour}\;\mathrm{VAF}/2\right)$$

Here binding score is 1/median neoantigen binding affinity, fold change is the difference in median binding affinity between neoantigen and wildtype peptide (agretopicity). The ranked tables of predicted neoantigens were then used as described in the Section 2.

### Immunopeptidomics

Snap frozen tissue samples were briefly thawed and weighed prior to 30 s of mechanical homogenization (Fisher, using disposable probes) in 4 ml lysis buffer (0.02 M Tris, 0.5% [w/v] IGEPAL, 0.25% [w/v] sodium deoxycholate, 0.15 mM NaCl, 1 mM ethylenediaminetetraacetic acid (EDTA), 0.2 mM iodoacetamide supplemented with EDTA‐free protease inhibitor mix). Homogenates were clarified for 10 min at 2000*g*, 4°C and then for a further 60 min at 13 500*g*, 4°C. Total 2 mg of anti‐major histocompatibility complex (MHC)‐I mouse monoclonal antibodies (W6/32) covalently conjugated to Protein A sepharose (Repligen) using Dimethyl pimelimidate as previously described [43, 44] were added to the clarified supernatants and incubated with constant agitation for 2 h at 4°C. The captured MHC‐I/β_2_m/immunopeptide complex on the beads was washed sequentially with 10 column volumes of low (isotonic, 0.15 M NaCl) and high (hypertonic, 0.4 M NaCl) Tris‐buffered saline washes prior to elution in 10% acetic acid and dried under vacuum. The MHC‐I‐depleted lysate was then incubated with anti‐MHC‐II mouse monoclonal antibodies (IVA12) and MHC‐II bound peptides were captured and eluted in the same conditions.Immunopeptides were separated from MHC‐I/β_2_m or MHC‐II heavy chain using offline high‐performance liquid chromatography (HPLC) on a C18 reverse phase column, as previously described [43]. Briefly, dried immunoprecipitates were reconstituted in buffer (1% acetonitrile, 0.1% trifluoroacetic acid) and applied to a 10 cm RP‐18e 100‐4.6 chromolith column (Merck) using an Ultimate 3000 HPLC equipped with UV monitor. Immunopeptides were then eluted using a 15 min 0%–40% linear acetonitrile gradient at a flow rate of 1 ml/min. Peptide fractions were eluted and pooled at between 0% and 30% acetonitrile, and the β_2_m and MHC heavy chains eluted at >40% acetonitrile.

HLA peptides were separated by an Ultimate 3000 RSLC nano system (Thermo Scientific) using a PepMap C18 EASY‐Spray LC column, 2 μm particle size, 75 μm x 75 cm column (Thermo Scientific) in buffer A (0.1% formic acid) and coupled on‐line to an Orbitrap Fusion Tribrid Mass Spectrometer (Thermo Fisher Scientific) with a nano‐electrospray ion source. Peptides were eluted with a linear gradient of 3%–30% buffer B (acetonitrile and 0.1% formic acid) at a flow rate of 300 nl/min over 110 min. Full scans were acquired in the Orbitrap analyser using the Top Speed data dependent mode, performing a MS scan every 3 s cycle, followed by higher energy collision‐induced dissociation (HCD) MS/MS scans. MS spectra were acquired at resolution of 120 000 at 300 *m*/*z*, Radio frequency lens 60% and an automatic gain control ion target value of 4.0e5 for a maximum of 100 ms. MS/MS resolution was 30 000 at 100 *m*/*z*. HCD fragmentation was induced at an energy setting of 28 for peptides with a charge state of 2–4, while singly charged peptides were fragmented at an energy setting of 32 at lower priority. Fragments were analysed in the Orbitrap at 30 000 resolution. Fragmented *m*/*z* values were dynamically excluded for 30 s.

### Proteomic data analysis

Raw spectrum files were analysed using Peaks Studio 10.0 build 20190129 [16, 45] and the data processed to generate reduced charge state and deisotoped precursor and associated product ion peak lists which were searched against the UniProt database (20 350 entries, 2020‐04‐07) plus the corresponding mutanome for each sample (~1000–5000 sequences) and contaminants list in unspecific digest mode. Parent mass error tolerance was set at 5 ppm and fragment mass error tolerance at 0.03 Da. Variable modifications were set for *N*‐term acetylation (42.01 Da), methionine oxidation (15.99 Da), carboxyamidomethylation (57.02 Da) of cysteine. As previously described, carbamidomethylated cysteines were treated as variable modifications due to the low concentration of 0.2 mM of iodoacetamide used in the lysis buffer to inhibit cysteine proteases [46]. A maximum of three variable modifications per peptide were set. The FDR was estimated with decoy‐fusion database searches [16] and were filtered to 1% FDR. Downstream analysis and data visualizations of the Peaks Studio identifications were performed in R using associated packages [42, 47].

### Immunopeptide HLA assignment

Identified immunopeptides were assigned to their HLA allotype for each patient using motif deconvolution tools and manual inspection. For class I HLA peptides initial assignment used MixMHCp (version 2.1) [7, 18] and for class II HLA peptides initial assignment used MoDec (version 1.1) [48]. Downstream analysis and data visualizations were performed in R using associated packages [17, 42, 47].

### Synthetic peptides

Peptides for functional T‐cell assays and spectra validation were synthesized using standard solid phase Fmoc chemistry (Peptide Protein Research Ltd., Fareham, UK).

### Functional T‐cell assay

PBMC (2 × 10^6^ per well) were stimulated in 24‐well plates with peptide (individual/pool) plus recombinant IL‐2 (R&D Systems Europe Ltd.) at a final concentration of 5 μg/ml and 20 IU/ml, respectively, and incubated at 37°C with 5% CO_2_; final volume was 2 ml. Media containing additional IL‐2 (20 IU/ml) was refreshed on Days 4, 6, 8 and 11 and on Day 13 cells were harvested. Expanded cells (1 × 10^5^ cell/well) were incubated in triplicate with peptide (individual) at 5 μg/ml final concentration for 22 h at 37°C in 5% CO_2_; phytohemagglutinin (Sigma‐Aldrich Company Ltd.) and CEFT peptide mix (JPT Peptide Technologies GmbH, Berlin, Germany), a pool of 27 peptides selected from defined HLA class I‐ and II‐restricted T‐cell epitopes, were used as positive controls. Spot forming cells were counted using the AID ELISpot plate reader system ELR04 and software (AID Autoimmun Diagnostika GmbH) and positivity calling for ELISpot data used the runDFR(×2) online tool (http://www.scharp.org/zoe/runDFR/). Downstream analysis and data visualizations were performed in R using associated packages [42, 47].

## Supporting information


**Appendix S1** Supporting information

## Data Availability

The mass spectrometry proteomics data have been deposited to the ProteomeXchange Consortium via the PRIDE [49] partner repository with the dataset identifier PXD031108 and 10.6019/PXD031108. Whole exome and RNA sequencing data have been deposited at the European Genome‐phenome Archive (EGA), which is hosted by the European Bioinformatics Institute and the Centre for Genomic Regulation, under accession number EGAS00001005957. The authors declare that all the other data supporting the finding of this study are available within the article and its supplementary information files and from the corresponding author on reasonable request.

## References

[1] Cancer Research UK . Oesophageal cancer statistics. United Kingdom: Cancer Research UK; 2022. Available:. https://www.cancerresearchuk.org/health-professional/cancer-statistics/statistics-by-cancer-type/oesophageal-cancer

[2] Smyth EC , Lagergren J , Fitzgerald RC , Lordick F , Shah MA , Lagergren P , et al. Oesophageal cancer. Nat Rev Dis Primers. 2017;3:1–21. 10.1038/nrdp.2017.48 PMC6168059 28748917

[3] Martínez‐Jiménez F , Muiños F , Sentís I , Deu‐Pons J , Reyes‐Salazar I , Arnedo‐Pac C , et al. A compendium of mutational cancer driver genes. Nat Rev Cancer. 2020;20:555–72. 10.1038/s41568-020-0290-x 32778778

[4] Secrier M , Li X , de Silva N , Eldridge MD , Contino G , Bornschein J , et al. Mutational signatures in esophageal adenocarcinoma define etiologically distinct subgroups with therapeutic relevance. Nat Genet. 2016;48:1131–41. 10.1038/ng.3659 27595477 PMC5957269

[5] Frankell AM , Jammula S , Li X , Contino G , Killcoyne S , Abbas S , et al. The landscape of selection in 551 esophageal adenocarcinomas defines genomic biomarkers for the clinic. Nat Genet. 2019;51:506–16. 10.1038/s41588-018-0331-5 30718927 PMC6420087

[6] Labani‐Motlagh A , Ashja‐Mahdavi M , Loskog A . The tumor microenvironment: a milieu hindering and obstructing antitumor immune responses. Front Immunol 2020;11 940. Available: 10.3389/fimmu.2020.00940 32499786 PMC7243284

[7] Bassani‐Sternberg M , Bräunlein E , Klar R , Engleitner T , Sinitcyn P , Audehm S , et al. Direct identification of clinically relevant neoepitopes presented on native human melanoma tissue by mass spectrometry. Nat Commun. 2016;7:13404. 10.1038/ncomms13404 27869121 PMC5121339

[8] Wells DK , van Buuren MM , Dang KK , Hubbard‐Lucey VM , Sheehan KCF , Campbell KM , et al. Key parameters of tumor epitope immunogenicity revealed through a consortium approach improve Neoantigen prediction. Cell. 2020;183:818–834.e13. 10.1016/j.cell.2020.09.015 33038342 PMC7652061

[9] Created with BioRender.com. BioRender; 2021. Available: https://biorender.com/.

[10] McGranahan N , Furness AJS , Rosenthal R , Ramskov S , Lyngaa R , Saini SK , et al. Clonal neoantigens elicit t cell immunoreactivity and sensitivity to immune checkpoint blockade. Science. 2016;351:1463–9. 10.1126/science.aaf1490 26940869 PMC4984254

[11] Yarchoan M , Hopkins A , Jaffee EM . Tumor Mutational Burden and Response Rate to PD‐1 Inhibition. 2017. Available: 10.1056/NEJMc1713444 PMC6549688 29262275

[12] Gori K , Baez‐Ortega A . sigfit: flexible Bayesian inference of mutational signatures. 2020 372896. doi:10.1101/372896.

[13] Alexandrov LB , Kim J , Haradhvala NJ , Huang MN , Tian Ng AW , Wu Y , et al. The repertoire of mutational signatures in human cancer. Nature. 2020;578:94–101. 10.1038/s41586-020-1943-3 32025018 PMC7054213

[14] Tate JG , Bamford S , Jubb HC , Sondka Z , Beare DM , Bindal N , et al. COSMIC: the catalogue of somatic mutations in cancer. Nucleic Acids Res. 2019;47:D941–7. 10.1093/nar/gky1015 30371878 PMC6323903

[15] Racle J , de Jonge K , Baumgaertner P , Speiser DE , Gfeller D . Simultaneous enumeration of cancer and immune cell types from bulk tumor gene expression data. eLife. 2017;6:e26476. 10.7554/eLife.26476 29130882 PMC5718706

[16] Zhang J , Xin L , Shan B , Chen W , Xie M , Yuen D , et al. PEAKS DB: De novo sequencing assisted database search for sensitive and accurate peptide identification. Mol Cell Proteomics. 2012;11:M111010587. 10.1074/mcp.M111.010587 PMC3322562 22186715

[17] Jessen LE . PepTools ‐ An R‐package for making immunoinformatics accessible. 2018. Available: https://github.com/leonjessen/PepTools.

[18] Gfeller D , Guillaume P , Michaux J , Pak H‐S , Daniel RT , Racle J , et al. The length distribution and multiple specificity of naturally presented HLA‐I ligands. J Immunol. 2018;201:3705–16. 10.4049/jimmunol.1800914 30429286

[19] Hundal J , Kiwala S , McMichael J , Miller CA , Xia H , Wollam AT , et al. pVACtools: a computational toolkit to identify and visualize cancer Neoantigens. Cancer Immunol Res. 2020;8:409–20. 10.1158/2326-6066.CIR-19-0401 31907209 PMC7056579

[20] Hundal J , Carreno BM , Petti AA , Linette GP , Griffith OL , Mardis ER , et al. pVAC‐seq: a genome‐guided in silico approach to identifying tumor neoantigens. Genome Med. 2016;8:11. 10.1186/s13073-016-0264-5 26825632 PMC4733280

[21] Izadi F , Sharpe BP , Breininger SP , Secrier M , Gibson J , Walker RC , et al. Genomic analysis of response to Neoadjuvant chemotherapy in esophageal adenocarcinoma. Cancer. 2021;13:3394. 10.3390/cancers13143394 PMC8308111 34298611

[22] Löffler MW , Mohr C , Bichmann L , Freudenmann LK , Walzer M , Schroeder CM , et al. Multi‐omics discovery of exome‐derived neoantigens in hepatocellular carcinoma. Genome Med. 2019;11:28. 10.1186/s13073-019-0636-8 31039795 PMC6492406

[23] Bobisse S , Genolet R , Roberti A , Tanyi JL , Racle J , Stevenson BJ , et al. Sensitive and frequent identification of high avidity neo‐epitope specific CD8^+^ T cells in immunotherapy‐naive ovarian cancer. Nat Commun. 2018;9:1092. 10.1038/s41467-018-03301-0 29545564 PMC5854609

[24] Rizvi NA , Hellmann MD , Snyder A , Kvistborg P , Makarov V , Havel JJ , et al. Mutational landscape determines sensitivity to PD‐1 blockade in nonsmall cell lung cancer. Science. 2015;348:124–8. 10.1126/science.aaa1348 25765070 PMC4993154

[25] Hilf N , Kuttruff‐Coqui S , Frenzel K , Bukur V , Stevanović S , Gouttefangeas C , et al. Actively personalized vaccination trial for newly diagnosed glioblastoma. Nature. 2019;565:240–5. 10.1038/s41586-018-0810-y 30568303

[26] Hu Z , Leet DE , Allesøe RL , Oliveira G , Li S , Luoma AM , et al. Personal neoantigen vaccines induce persistent memory T cell responses and epitope spreading in patients with melanoma. Nat Med. 2021;27:515–25. 10.1038/s41591-020-01206-4 33479501 PMC8273876

[27] Wang M , Larsen MV , Nielsen M , Harndahl M , Justesen S , Dziegiel MH , et al. HLA class I binding 9mer peptides from influenza a virus induce CD4^+^ T cell responses. PLOS ONE. 2010;5:e10533. 10.1371/journal.pone.0010533 20479886 PMC2866539

[28] Ott PA , Hu Z , Keskin DB , Shukla SA , Sun J , Bozym DJ , et al. An immunogenic personal neoantigen vaccine for patients with melanoma. Nature. 2017;547:217–21. 10.1038/nature22991 28678778 PMC5577644

[29] Arnaud M , Chiffelle J , Genolet R , Navarro Rodrigo B , Perez MAS , Huber F , et al. Sensitive identification of neoantigens and cognate TCRs in human solid tumors. Nat Biotechnol. 2021;40:1–5. 10.1038/s41587-021-01072-6 PMC9110298 34782741

[30] Danecek P , Bonfield JK , Liddle J , Marshall J , Ohan V , Pollard MO , et al. Twelve years of SAMtools and BCFtools. GigaScience. 2021;10. 10.1093/gigascience/giab008 PMC7931819 33590861

[31] Picard toolkit. Broad Institute; 2019. Available: http://broadinstitute.github.io/picard/

[32] Van der Auwera GA , O'Connor BD . Genomics in the cloud. O'Reilly Media, Inc. ISBN: 9781491975190. Available: https://www.oreilly.com/library/view/genomics‐in‐the/9781491975183/

[33] Benjamin D , Sato T , Cibulskis K , Getz G , Stewart C , Lichtenstein L . Calling somatic SNVs and Indels with Mutect2. bioRxiv. 2019;861054. 10.1101/861054

[34] Koboldt DC , Zhang Q , Larson DE , Shen D , McLellan MD , Lin L , et al. VarScan 2: Somatic mutation and copy number alteration discovery in cancer by exome sequencing. Genome Res. 2012;22:568–76. 10.1101/gr.129684.111 22300766 PMC3290792

[35] Kim S , Scheffler K , Halpern AL , Bekritsky MA , Noh E , Källberg M , et al. Strelka2: fast and accurate calling of germline and somatic variants. Nat Methods. 2018;15:591–4. 10.1038/s41592-018-0051-x 30013048

[36] Bonfield JK , Marshall J , Danecek P , Li H , Ohan V , Whitwham A , et al. HTSlib: C library for reading/writing high‐throughput sequencing data. GigaScience. 2021;10:giab007. 10.1093/gigascience/giab007 33594436 PMC7931820

[37] McLaren W , Gil L , Hunt SE , Riat HS , Ritchie GRS , Thormann A , et al. The ensembl variant effect predictor. Genome Biol. 2016;17:122. 10.1186/s13059-016-0974-4 27268795 PMC4893825

[38] Chen S , Zhou Y , Chen Y , Gu J . Fastp: An ultra‐fast all‐in‐one FASTQ preprocessor. Bioinformatics. 2018;34:i884–90. 10.1093/bioinformatics/bty560 30423086 PMC6129281

[39] Kim D , Paggi JM , Park C , Bennett C , Salzberg SL . Graph‐based genome alignment and genotyping with HISAT2 and HISAT‐genotype. Nat Biotechnol. 2019;37:907–15. 10.1038/s41587-019-0201-4 31375807 PMC7605509

[40] Kovaka S , Zimin AV , Pertea GM , Razaghi R , Salzberg SL , Pertea M . Transcriptome assembly from long‐read RNA‐seq alignments with StringTie2. Genome Biol. 2019;20:278. 10.1186/s13059-019-1910-1 31842956 PMC6912988

[41] Hundal J , Kiwala S , Feng Y‐Y , Liu CJ , Govindan R , Chapman WC , et al. Accounting for proximal variants improves neoantigen prediction. Nat Genet. 2019;51:175–9. 10.1038/s41588-018-0283-9 30510237 PMC6309579

[42] R Core Team : R: A language and environment for statistical computing. Vienna, Austria: R Foundation for Statistical Computing; 2018. Available: https://www.R-project.org/

[43] Purcell AW , Ramarathinam SH , Ternette N . Mass spectrometrybased identification of MHC‐bound peptides for immunopeptidomics. Nat Protoc. 2019;14:1687–707. 10.1038/s41596-019-0133-y 31092913

[44] Bailey A , Nicholas B , Darley R , Parkinson E , Teo Y , Aleksic M , et al. Characterization of the class I MHC Peptidome resulting from DNCB exposure of HaCaT cells. Toxicol Sci. 2020;180:136–47. 10.1093/toxsci/kfaa184 PMC7916740 33372950

[45] Tran NH , Zhang X , Xin L , Shan B , Li M . De novo peptide sequencing by deep learning. Proc Natl Acad Sci USA. 2017;114:8247–52. 10.1073/pnas.1705691114 28720701 PMC5547637

[46] Chong C , Marino F , Pak H , Racle J , Daniel RT , Müller M , et al. High‐throughput and sensitive Immunopeptidomics platform reveals profound Interferonγ‐mediated remodeling of the human leukocyte antigen (HLA) ligandome*. Mol Cell Proteom. 2018;17:533–48. 10.1074/mcp.TIR117.000383 PMC5836376 29242379

[47] Wickham H , Averick M , Bryan J , Chang W , McGowan LD , François R , et al. Welcome to the tidyverse. J Open Source Softw. 2019;4:1686. 10.21105/joss.01686

[48] Racle J , Michaux J , Rockinger GA , Arnaud M , Bobisse S , Chong C , et al. Robust prediction of HLA class II epitopes by deep motif deconvolution of immunopeptidomes. Nat Biotechnol. 2019;37:1283–6. 10.1038/s41587-019-0289-6 31611696

[49] Perez‐Riverol Y , Csordas A , Bai J , Bernal‐Llinares M , Hewapathirana S , Kundu DJ , et al. The PRIDE database and related tools and resources in 2019: improving support for quantification data. Nucleic Acids Res. 2019;47:D442–50. 10.1093/nar/gky1106 30395289 PMC6323896

